# Beyond Cancer Cells: How the Tumor Microenvironment Drives Cancer Progression

**DOI:** 10.3390/cells13191666

**Published:** 2024-10-09

**Authors:** Hussein Sabit, Borros Arneth, Shaimaa Abdel-Ghany, Engy F. Madyan, Ashraf H. Ghaleb, Periasamy Selvaraj, Dong M. Shin, Ramireddy Bommireddy, Ahmed Elhashash

**Affiliations:** 1 Department of Medical Biotechnology, College of Biotechnology, Misr University for Science and Technology, Giza P.O. Box 77, Egypt; hussein.sabit@must.edu.eg (H.S.); engy.madyan@must.edu.eg (E.F.M.); 2 Institute of Laboratory Medicine and Pathobiochemistry, Molecular Diagnostics, Hospital of the Universities of Giessen and Marburg (UKGM), Philipps University Marburg, Baldinger Str., 35043 Marburg, Germany; 3 Institute of Laboratory Medicine and Pathobiochemistry, Molecular Diagnostics, Hospital of the Universities of Giessen and Marburg (UKGM), Justus Liebig University Giessen, Feulgenstr. 12, 35392 Giessen, Germany; 4 Department of Environmental Biotechnology, College of Biotechnology, Misr University for Science and Technology, Giza P.O. Box 77, Egypt; shaimaa.ibraheem@must.edu.eg; 5 Department of Surgery, College of Medicine, Misr University for Science and Technology, Giza P.O. Box 77, Egypt; ashraf.ghaleb@must.edu.eg; 6 Department of Surgery, College of Medicine, Cairo University, Giza 12613, Egypt; 7 Department of Pathology and Laboratory Medicine, Emory University School of Medicine, Atlanta, GA 30322, USA; pselvar@emory.edu (P.S.); ramireddy.bommireddy@emory.edu (R.B.); 8 Department of Hematology and Medical Oncology, Emory University School of Medicine, Atlanta, GA 30322, USA; dmshin@emory.edu; 9 Department of Biology, Texas A&M University, 3258 TAMU I, College Station, TX 77843-3258, USA

**Keywords:** tumor microenvironment, TME, ECM, liver cancer, immunotherapy, targeted therapy

## Abstract

Liver cancer represents a substantial global health challenge, contributing significantly to worldwide morbidity and mortality. It has long been understood that tumors are not composed solely of cancerous cells, but also include a variety of normal cells within their structure. These tumor-associated normal cells encompass vascular endothelial cells, fibroblasts, and various inflammatory cells, including neutrophils, monocytes, macrophages, mast cells, eosinophils, and lymphocytes. Additionally, tumor cells engage in complex interactions with stromal cells and elements of the extracellular matrix (ECM). Initially, the components of what is now known as the tumor microenvironment (TME) were thought to be passive bystanders in the processes of tumor proliferation and local invasion. However, recent research has significantly advanced our understanding of the TME’s active role in tumor growth and metastasis. Tumor progression is now known to be driven by an intricate imbalance of positive and negative regulatory signals, primarily influenced by specific growth factors produced by both inflammatory and neoplastic cells. This review article explores the latest developments and future directions in understanding how the TME modulates liver cancer, with the aim of informing the design of novel therapies that target critical components of the TME.

## 1. Introduction 

**Figure 1:**
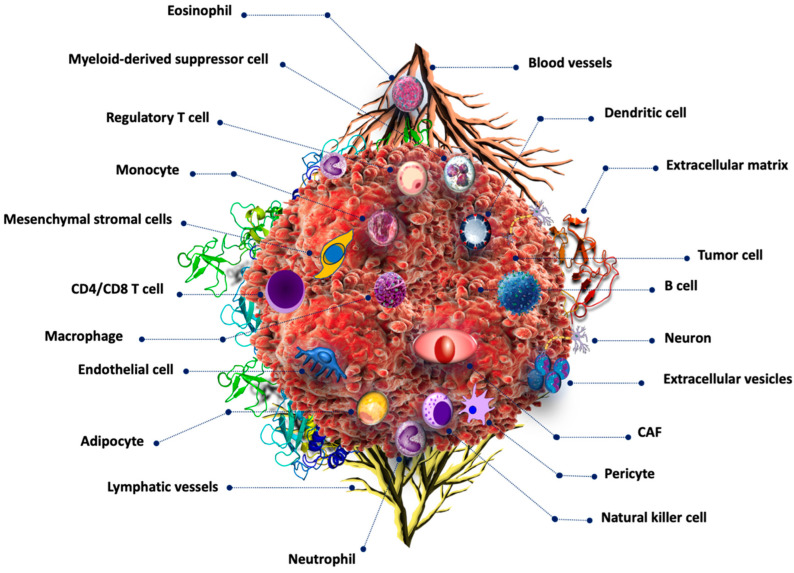
Tumor microenvironment components. Cancer cells are surrounded by numerous non-cancerous cells including those related to the immune system such as B cells, T-cells, dendritic cells, monocytes, eosinophils, and basophils, among others. Cancer-associated fibroblasts are also common in the TME.

Cancer development is a multistep process regulated by various intrinsic and extrinsic factors, such as mutations in tumor suppressor genes, protooncogenes, infections, environmental factors, diet, and lifestyle. Tumor growth is supported by various types of non-cancerous cells in the tumor microenvironment (TME). The TME is critical for tumor growth and progression. The TME is a dynamic system composed of immunological, neoplastic, stromal, and extracellular matrix cells [1]. Figure 1 depicts the main components of the TME. The TME is highly immunosuppressive and supports cancer growth and metastasis [2,3]. 

Hypoxia is common in solid tumors, including liver cancer, and it induces hypoxia-inducible factors (HIFs). HIF signaling in innate immune cells and cancer cells activates pro-tumorigenic immune cells and inhibits anti-tumor immune cells, enabling immune evasion [4]. Thus, HIFs are potential therapeutic targets for decreasing immunosuppression and cancer progression. Cancer-associated fibroblasts (CAF) and the extracellular matrix (ECM) protect cancer cells from immune surveillance, as well as from therapeutic antibodies and drugs. CAFs also promote tumor growth by secreting tumor-promoting and immune-suppressing cytokines, chemokines, and growth factors and contribute to resistance to chemotherapy and immunotherapy [5]. 

Herein, we describe the components of TME and review their unique roles in cancer. Furthermore, the roles of hypoxia, immune cells, and ECM in immune invasion and future directions are also discussed.

## 2. Tumor Microenvironment

The tumor microenvironment is particularly challenging for immune cell infiltration, activation, and effector function due to its acidic pH, hypoxic conditions, and limited nutrient availability. Tumor cells predominantly rely on aerobic glycolysis for their proliferation; however, there is significant heterogeneity in the glycolytic and oxidative capacities among different tumor cells [6]. Initially, it was thought that glycolysis served as the primary mechanism for ATP production in tumors due to the belief that cancer cells possessed impaired mitochondria. However, recent findings have demonstrated that mitochondria in cancer cells are not merely passive entities but are actively involved in processes beyond conventional mitochondrial respiration [7]. Given the high energy demands of rapidly proliferating tumor cells, they utilize both aerobic glycolysis and oxidative phosphorylation, depleting essential nutrients from the surrounding environment. This results in a tumor microenvironment characterized by low glucose levels, hypoxia, and acidic pH [8].

### 2.1. Extracellular pH

**Figure 2:**
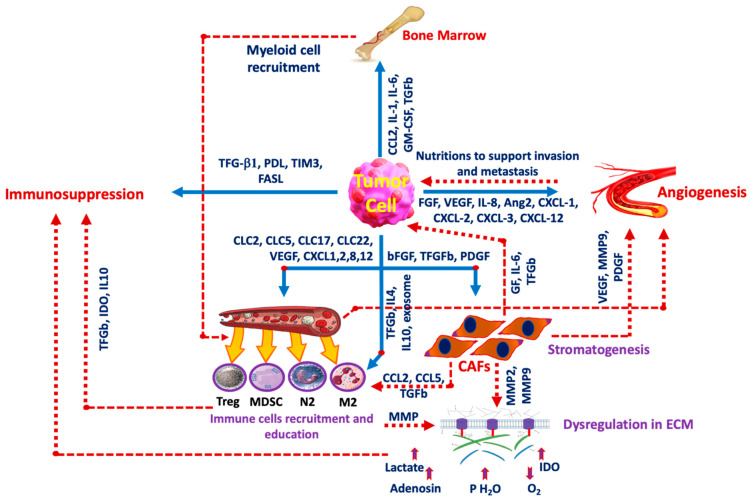
Tumor cell interaction with the microenvironment. The interplay between cancer cells and The TME is the main factor in cancer progression. Some inducers are released from the tumor cells to affect other components such as the FGF family, VEGF, and IL-8 among others which induce angiogenesis, and other factors including TGF-b1 and FASL induce immunosuppression. Tumor cells also release IL-4 and IL-19 to induce immune cell recruitment and education and release CCL2, IL-1, and IL-6 to activate bone marrow to take part in the production of myeloid cell recruitment. GM-CSF: Granulocyte-macrophage colony-stimulating factor, FGF: Fibroblast growth factor, Ang2: Angiopoietin-2, PDGF: Platelet-derived growth factor, PD-L1: Programmed Cell Death Ligand-1, TIM3: T-cell immunoglobulin domain and mucin domain 3, FASL: Fas ligand, CXCL: chemokines, CAF: cancer-associated fibroblasts, IL: Interleukin, M2: M2 macrophage, N2: Neutrophil, Treg: T-regulatory lymphocyte, MDSC: myeloid-derived suppressor cell, MMP: Matrix metalloproteinase, GF: Growth factor, IDO: Indolamine 2,3 dioxygenase.

Cancer’s extensive metabolic reprogramming is complex and may involve metabolic cooperation between cancer cells and the surrounding stroma [9]. One key aspect of this reprogramming is the acidity of the tumor microenvironment (TME), which has been a focal point in research on cancer-related metabolic changes. Acidosis plays a critical role in the development of malignancy and somatic evolution [10]. It influences malignant behavior, metastasis, and invasion rates, and impacts the immunosurveillance mechanism by inducing the polarization of tumor-associated macrophages (TAMs) to the M2 phenotype under hypoxic conditions [11]. In poorly oxygenated regions, such as the tumor core, cells shift their energy production from the efficient, oxygen-dependent oxidative phosphorylation to the less efficient, oxygen-independent anaerobic glycolysis. This shift leads to a buildup of lactic acid, creating a more acidic environment within the tumor [12]. Acidosis is no longer considered a mere byproduct of tumor growth; it is now recognized as a critical regulator of tumor progression, closely linked to extracellular lactic acid accumulation and hypoxia [13]. The hyperglycolytic phenotype of cancer can lead to an accumulation of lactate and protons, which must be expelled into the extracellular environment to maintain intracellular pH and essential cellular functions. As a result, cancer cells exhibit a higher intracellular pH compared to healthy cells, while their extracellular pH is more acidic than that of normal tissues [14]. Extracellular tumor acidosis is associated with aggressive tumor growth and invasion, neoangiogenesis, and metastasis [15]. Elevated levels of carbonic anhydrases, monocarboxylate transporters 1 and 4, and the Na^+^-H^+^ exchanger 1 in tumors facilitate the release of protons and lactate into the extracellular environment [16]. The acidic extracellular pH in the TME can serve as a biomarker for oncologic imaging to detect the effects of increased glycolysis and as a therapeutic target to overcome resistance mechanisms to chemotherapy or radiation. Numerous studies have linked tumor acidity to many aspects of cancer growth and progression, including distant metastatic spread and local tumor invasion. Researchers have recently discovered that lowering the pH in the TME might promote cancer cell motility and create changes in cytoskeletal dynamics that affect macrophage and fibroblast polarization and function [13] (Figure 2).

One of the key metabolites produced during glycolysis is lactate, which is transported out of the cell by monocarboxylate transporters (MCTs) [17]. Elevated levels of lactate and kynurenine in the TME are known to enhance the immunosuppressive functions of regulatory T-cells, enabling them to dominate over CD8 effector cells [18]. Additionally, studies have shown that the acidification caused by the efflux of lactic acid and protons suppresses the production of inflammatory cytokines in CD8 T-cells and natural killer (NK) cells, thereby inhibiting NFAT activation and reducing cytotoxicity [19]. Inhibitors of MCTs have been employed to block lactate export from tumor cells, thereby reducing acidosis within the TME. AZD3965, a potent MCT1 inhibitor currently in clinical trials (NCT01791595), has been shown to inhibit the growth of various cell lines in culture and to limit glycolysis by causing an accumulation of intracellular lactate [20]. Recent studies have further demonstrated that combining metformin with the inhibition of lactate transporters MCT1 and MCT4 is synthetically lethal for cancer cells in culture [21]. Figure 2 illustrates the interaction between the TME and cancer cells.

LAMP2 (lysosome-associated membrane protein 2) plays a crucial role in helping cancer cells survive in acidic environments [15]. It functions by protecting lysosomal membranes from acidic degradation during cancer progression. The increased acidity in the TME can trigger the induction of the autophagy regulator autophagy-related gene 5 (ATG5) in pre-invasive cancer cells [22]. Additionally, cells that are exposed to low pH conditions for prolonged periods exhibit elevated levels of autophagy markers such as ATG5 and BNIP3, a member of the BCL-2 family [23]. Autophagy plays a dual role in cancer, promoting tumor survival by recycling damaged components under stress, while in other contexts, it can suppress tumor growth by removing harmful mutations and leading to autophagic cell death [24,25]. Despite these observations, the precise mechanisms underlying these changes remain largely unknown. Tumor acidosis is increasingly recognized as a promising therapeutic target for developing new cancer treatments [26]. When designing strategies to target tumor acidosis, it is crucial to consider the metabolic vulnerabilities associated with acidosis, the potential for neutralizing acidity using buffers, and approaches to inhibit hydrogen ion production. 

### 2.2. Hypoxia

Hypoxia is a critical element of the tumor microenvironment (TME), arising from uncontrolled proliferation and poor vascularization, which leaves tumors utilizing partial metabolic respiration in a highly oxygen-deprived environment. Oxygen levels within tumors are often heterogeneous, leading to varied responses to immunotherapy across different tumor regions [27]. Hypoxia can suppress T-cell receptor (TCR) signaling and increase the prevalence of regulatory T-cells [28]. Interestingly, metastases due to a lack of a robust immune response have been found to exhibit increased oxidative phosphorylation pathways. Inhibiting mitochondrial respiration in such cases has been shown to improve survival in both murine tumor implantation models and spontaneous brain metastasis models [29]. While the impact of tumor glycolysis on limiting anti-tumor responses is well-documented [30], the roles of hypoxia and mitochondrial respiration are emerging as promising therapeutic targets.

Hypoxia contributes to tumor progression by promoting endothelial-to-mesenchymal transition (EMT) and facilitating metastasis through the upregulation of growth factors, such as hypoxia-inducible factor (HIF)-1 [31,32]. Moreover, hypoxia fosters the expression of immune checkpoints, activates regulatory T-cells, and polarizes macrophages toward an anti-inflammatory, pro-tumorigenic M2 phenotype. Hypoxia is a pervasive, continuous, and complex condition affecting both malignant and stromal cells. It is frequently associated with poor prognosis and contributes to cancer progression by influencing several aspects of cancer biology, including tumor growth, stemness, dormancy, redox adaptation, intercellular communication, and resistance to therapy [33]. Cancer cells depend heavily on the upregulation of hypoxia-inducible factors (HIFs) and HIF signaling to adapt to low-oxygen environments. Intra-tumoral hypoxia results from the imbalance between cancer cell expansion and oxygen supply, which is further compounded by metabolic shifts within the cancer cells. Additionally, hypoxia drives angiogenesis by increasing VEGF production and activating vascular endothelial cells, all of which significantly influence the TME and the effectiveness of therapies [34,35].

The genes SLC2A1, VEGFA, ENO1, LDHA, TUBB6, ALDOA, TP11, ADM, NDRG1, MIF, P4HA1, MRPS1, CDKN3, PGAM1, and ACOT7 are among the top-ranked hypoxia-associated genes [36,37,38]. In their exploration of hypoxia in cancer, Bhandari et al. [39] analyzed 1188 tumors from 27 categories, encompassing both solid and hematologic malignancies. Their findings reveal that hypoxia displays both intra- and inter-tumor heterogeneity among different cancer types and even among patients with the same type of cancer. For instance, lung and cervical squamous cell carcinomas are among the most hypoxic cancer types, while thyroid adenocarcinoma and chronic lymphocytic leukemia exhibit the lowest hypoxia scores [40]. Moreover, the hypoxic niche is associated with a higher mutational burden of somatic variants and alterations in key oncogenes and tumor suppressors, including TP53, PTEN, and MYC [32,41].

The acidic niche is closely connected with the hypoxic niche and the broader metabolic microenvironment, particularly in relation to lactate metabolism, which plays a key role in its formation through processes such as lactate production and CO_2_ hydration [42,43,44]. Hypoxia can drive increased lactate production and proton accumulation in hypoxic zones, leading to the acidification of the TME and enhancing the adaptability of tumor cells [42]. This acidic environment not only facilitates tumor invasion and metastasis but also interacts with lactate metabolism to establish a favorable milieu for cancer progression. This interplay, termed lactate-based metabolic symbiosis, was first recognized in this context. Notably, the acidic microenvironment has been shown to enhance oxidative phosphorylation, EMT, and the invasiveness of melanoma cells [45]. Imaging hypoxia within the TME prior to therapy could potentially help in identifying and assessing the tumor’s hypoxic state and monitoring changes induced by treatment. However, hypoxia imaging techniques have yet to be integrated into clinical practice.

### 2.3. ROS in TME 

Reactive oxygen species (ROS) are tightly regulated in healthy cells, acting as secondary messengers in response to various environmental stimuli. However, in tumor cells, aberrant ROS accumulation and signaling cascades contribute to the oncogenic phenotype. ROS not only impacts tumor epithelial cells but also influences the surrounding cells in the tumor microenvironment (TME). Elevated ROS levels trigger inflammation, promote the differentiation of fibroblasts into myofibroblasts, and enhance tumor angiogenesis. Chronic oxidative stress significantly alters the function of these fibroblast subtypes, driving tumor growth and metastatic spread [46]. In the TME, ROS plays a pivotal role in maintaining redox homeostasis and regulating oxidative stress. Due to the TME’s capacity for rapid proliferation and its unique metabolic patterns, ROS are involved in nearly all complex physiological processes, influencing protein alteration, signal transduction, metabolism, and energy production across various tumors [47].

Understanding the dynamic and multicomponent changes in ROS within the TME is crucial for elucidating the specific mechanisms underlying tumor proliferation and metastasis [48]. Additionally, ROS are linked to tumor-induced immunosuppression and serve as critical signaling mediators within the immune system. They are regulated by cytokines, amino acid metabolism, and enzymatic activity [49]. By releasing ROS, immunosuppressive cells accumulate in the TME, leading to T-cell apoptosis and functional suppression. Consequently, controlling ROS levels may be key to prolonging T-cell survival and enhancing antitumor activity [49]. 

Notably, elevated ROS levels have been associated with carcinogenesis, tumor immunity, and the reprogramming of the TME [50]. Under hypoxic conditions, mitochondrial ROS stabilize hypoxia-inducible factors (HIF), which may promote autophagy and enhance tumorigenicity [51,52]. Both tumor and stromal cells within the TME generate ROS, which can influence cancer cell development. As cancer cells evolve, they become tolerant to ROS accumulation, a condition termed ROS addiction [53]. 

Furthermore, ROS play a role in the renewal of cancer stem cells (CSCs) and the epithelial–mesenchymal transition (EMT), contributing to drug resistance in various cancers, including liver cancer [54]. The immunosuppression linked to increased ROS production can undermine the ability of immune cells to control tumor growth [55]. Therefore, targeting ROS represents a potential strategy for cancer prevention and treatment. However, the challenge lies in the dual role of ROS; while elevated levels can promote antioxidant capacity and redox balance, they can also drive tumor growth and metastasis. The sources and functions of ROS may vary at different stages of tumor development, making the heterogeneous TME a complex target for therapeutic intervention [56].

### 2.4. TME Reprogramming

The TME encompasses immune cells that engage in both anti-tumor and tumor-promoting interactions with stromal and non-tumor cells. Enhancing treatment efficacy can be achieved by modifying the tumor’s immune microenvironment, making any tumor a viable target through TME reprogramming [57,58]. Various strategies have been suggested, including photodynamic therapy (PDT) and nitric oxide (NO) therapy, which, when combined, amplify the antitumor immune response [59].

The hypoxic niche pervades nearly the entire tumor and its surrounding environment, triggering a hypoxia-induced cascade that affects not only cancer cells but also other specialized microenvironments within the TME. Notably, the immune system, lactate and ROS metabolism, and the acidic niche are significantly impacted. According to the theories of metabolic symbiosis and the glycolytic switch, oxidative cancer cells favor lactate over glucose, with MCT1 facilitating lactate exchange in tumors [60,61,62,63]. In this process, hypoxic cells utilize glucose to produce lactate, which diffuses according to the concentration gradient, while oxidative cancer cells absorb lactate via MCT1.

Cancer-associated fibroblasts (CAFs) mediate bidirectional communication between mechanical and hypoxic environments [64,65,66]. They play a key role in extracellular matrix (ECM) remodeling and the creation of a hypoxic microenvironment. For instance, in breast cancer, CAFs become elongated and spindle-shaped, secrete more type I collagen, enhance matrix adherence and mesenchymal morphology, and produce ECM with increased stiffness and aligned collagen fibers, thereby facilitating cancer cell invasion and migration [66,67]. CAFs are also crucial in the TME of cancers such as the liver and pancreas, where they support tumor growth and are associated with poorer outcomes [68]. Despite their potential as therapeutic targets, the diversity of CAFs and the lack of specific markers present challenges in developing effective cancer treatments [69].

Understanding and recapturing the hypoxic niche while exploring these mechanisms could enhance fundamental research and aid in translating strategies into clinical settings, ultimately leading to better outcomes with novel therapeutic approaches (reviewed in [70]).

### 2.5. Extracellular Matrix 

The extracellular matrix (ECM) is a non-cellular tissue component that provides crucial structural and biochemical support to cells. It possesses physiological properties similar to those of living cells [71], influencing cell communication, adhesion, and proliferation rather than merely serving as an intercellular filler. The ECM is composed of water, fibrous proteins, proteoglycans, and minerals [72,73,74]. The unique composition of the ECM is attributed to the biochemical and biophysical interactions between cellular components and the microenvironment where tissues develop. ECM components vary depending on the resident cells and the specific needs of the tissue. Recent studies have shown that the ECM plays a significant role in the formation of various fibrous tissues [75]. 

Cancer cells secrete several growth factors, including platelet-derived growth factor (PDGF), transforming growth factor-beta (TGF-β), VEGF, basic fibroblast growth factor (bFGF), and interleukins, which regulate the TME [76]. The increased expression of these mediators often leads to the production of proteolytic enzymes by tumor cells, exerting autocrine effects and triggering the release of these substances from stromal cells, such as fibroblasts.

Glucose, in addition to being an energy source, contributes to ECM formation, providing structural and biochemical support to tumor cells and other cells within the TME [77,78]. Along with collagen and elastin, proteoglycans, a subclass of glycoproteins, constitute a significant portion of the ECM. These are connected to a core protein by at least one glycosaminoglycan chain and are found in the cytoplasm, on the cell surface, and within the ECM [79]. The connection between tumor growth and ECM is described in detail in the work of Arneth [80]. 

Several tumors, particularly liver cancer, express high levels of Glypican-3 (GPC-3), a marker associated with poor prognosis. GPC-3, an oncofetal protein overexpressed in lung, breast, colon, liver, and head and neck cancers, is a promising drug target [81]. Its roles in cancer progression include recruiting and polarizing macrophages toward an M2 phenotype, promoting EMT, and stimulating Wnt signaling to advance tumor growth [82,83,84]. Anti-GPC-3 chimeric antigen receptor (CAR) T-cells, combined with soluble IL-15, have shown significant efficacy in preclinical mouse models of hepatocellular carcinoma (HCC), highlighting GPC-3’s therapeutic potential. Due to its high tumor-to-liver ratio, GPC-3 is also ideal for targeted imaging in liver cancer.

The breakdown of ECM and cell surface proteins is critically dependent on matrix metalloproteinases (MMPs), bone morphogenic protein 1 (BMP1), tissue serine proteinases, and adamalysin-related membrane proteinases [85]. A recent study demonstrated that both stromal and tumor cells can modify the ECM to create an environment conducive to tumor cell microinvasion [86]. Modifications to the ECM can release new molecular fragments and expose cryptic protein sites, which significantly impact migratory and angiogenic properties. For example, fibronectin, a component of the ECM, contains cryptic protein domains that are often folded and hidden. Proteolytic enzymes can expose these domains, revealing new integrin-binding sites and anti-angiogenic sequences [87].

Hyaluronan, a major glycosaminoglycan in the ECM, promotes cell proliferation and migration [88,89]. It also increases ECM tension in the TME [90], leading to growth-induced solid stress and significant mechanical load on blood vessels. Lymphatic endothelial cells uniquely express the hyaluronan receptor LYVE-1 [91], which induces lymphangiogenesis and angiogenesis in tumors. The role of LYVE-1 receptors in tumor-associated lymphangiogenesis remains unclear. However, recent studies have shown that low molecular weight hyaluronan can bind to LYVE-1, promoting lymphatic endothelial cell migration, proliferation, and tube formation [92,93]. Dynamic ECM remodeling and the interaction between cells and their substrates are essential components of tumor-mediated lymphangiogenesis.

Research has also shown that the ECM can function as a reservoir for TGF-β [94]. Typically, TGF-β regulates epithelial cell growth, and mutations in TGF-β signaling molecules, such as TGF-βRII and SMAD4, are associated with tumor formation in the gastrointestinal tract [95]. However, in hepatocellular carcinoma, SMAD4, a common SMAD for TGF-β and BMP signaling, has been shown to promote tumor growth via a non-canonical signaling mechanism [96]. TGF-β is crucial for maintaining tissue homeostasis and preventing tumor formation in nearly all human cells. However, genetically unstable cancer cells can evade the tumor-suppressive effects of the TGF-β pathway in the TME. They achieve this by deactivating key components of the TGF-β pathway, such as TGF-β receptors, or by inhibiting other components that suppress tumor growth [97]. Tumors that produce TGF-β induce immune suppression by converting T-cells to regulatory T-cells (Tregs), promoting the expansion of myeloid-derived suppressor cells (MDSCs), and polarizing macrophages to an M2 phenotype. Tumor-secreted TGF-β1 also recruits stromal cells, such as myofibroblasts and osteoclasts, which contribute to tumorigenesis.

The composition and biomechanical properties of the ECM influence integrin signaling, which affects key cancer formation processes, including the Hippo pathway and EMT [97]. Integrins, which cells use to bind to the ECM, are crucial for promoting epithelial differentiation and cell development [98]. Additionally, the loss of integrin subunits, such as β2 and β6, may accelerate tumor growth. Syndecans, which bind ECM proteins such as collagen and laminin, are necessary for integrin function and activity.

## 3. Inflammation and Immune Evasion

**Figure 3:**
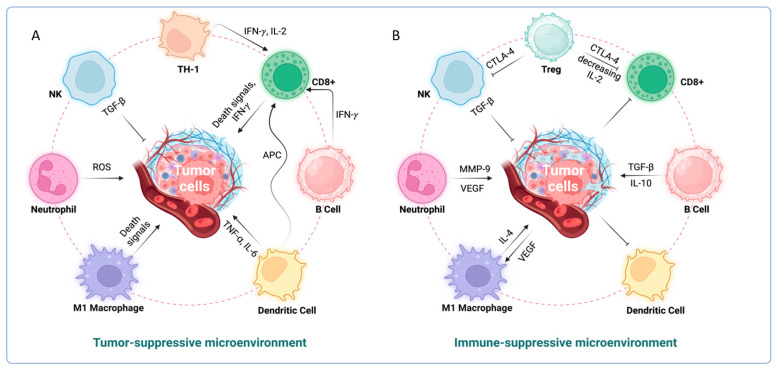
Immunological mechanisms regulating tumor growth. (**A**) Tumor suppressive microenvironment. Tumor cell proliferation is inhibited by activated CD4+, CD8+, NK, M1 macrophages, and neutrophils. (**B**) Immunosuppressive microenvironment. Tumor cells that secrete factors such as TGFβ1, G-CSF, etc. promote MDSC, Treg cells, and M2 macrophages, which inhibit anti-tumor T-cells and NK cells.

The tumor microenvironment (TME) in liver cancer is composed of hepatic stellate cells, endothelial cells, and neighboring immune cells. The TME creates a hostile setting for immune cell infiltration due to the presence of dense stroma and competition for nutrients, leading to immune suppression and tumor advancement. Cancer cells heavily rely on aerobic glycolysis for energy. Similarly, proinflammatory M1 macrophages and activated T-cells shift their metabolism to an active state, enhancing nutrient absorption, glutaminolysis, and aerobic glycolysis (oxidative phosphorylation) to fuel their effector functions [99]. However, in the TME, cancer cells often outcompete local immune cells for nutrients, thereby gaining a survival advantage [100] (Figure 3).

Inflammatory stimuli polarize macrophages toward an M1-like phenotype, resulting in the production of pro-inflammatory cytokines [101]. Conversely, anti-inflammatory stimuli induce polarization toward an M2-like phenotype, which has immunosuppressive properties. Prolonged inflammation, such as that seen in chronic viral hepatitis, increases the prevalence of immunosuppressive M2-like macrophages [102,103]. Tumor-derived lactate plays a crucial role in this process by enhancing VEGF expression and promoting M2 polarization of TAMs, which in turn foster angiogenesis and immunosuppression [104]. Moreover, lactate accumulation and the acidic TME reduce interferon-gamma expression in CD8 T-cells and NK cells, further weakening the immune response [105,106]. M2-like TAMs are also associated with poor overall survival in hepatocellular carcinoma (HCC) and can drive EMT and chemoresistance [107]. These findings underscore the importance of targeting the immune microenvironment with novel therapeutic strategies to improve patient outcomes.

## 4. Cancer-Associated Fibroblasts (CAFs)

There is increasing evidence that fibroblasts play a crucial role in cancer progression [108,109]. Cancer-associated fibroblasts (CAFs) are spindle-shaped mesenchymal cells, sharing characteristics with both fibroblasts and smooth muscle cells [110], though they originate from different sources. Immunohistochemically, CAFs can be distinguished from typical stromal fibroblasts by their higher levels of smooth muscle actin, vimentin, desmin, and fibroblast-activating protein (FAP), using a combination of these markers [111]. Within the tumor microenvironment (TME), CAFs are heterogeneous, exhibiting diverse origins, functions (either pro-tumor or anti-tumor), and surface markers such as alpha-smooth muscle actin (α-SMA), myosin light chain 9 (MYL9), myosin light chain kinase (MYLK), matrix metalloproteinase 2 (MMP2), decorin (DCN), and collagen type I alpha 2 (COL1A2) [112,113].

Cancer-associated fibroblasts (CAFs) play a pivotal role in the tumor microenvironment (TME), contributing significantly to tumor formation and progression through the synthesis of growth factors, cytokines, extracellular matrix (ECM) proteins (including collagen and fibronectin), and matrix metalloproteinases (MMPs) [114]. Notably, CAFs exhibit elevated levels of transforming growth factor-beta 1 (TGF-β1), insulin-like growth factor-binding protein 2, tumor necrosis factor superfamily member 4, and heparin-binding EGF-like growth factor compared to normal fibroblasts [115,116]. CAFs also secrete growth factors such as hepatocyte growth factor (HGF) and ECM glycoproteins such as tenascin-C (TNC). Conversely, tumor cells produce TGF-β1 and platelet-derived growth factor (PDGF), which are critical in mediating interactions between tumors and fibroblasts. TGF-β1 induces the differentiation of fibroblasts and myofibroblasts into CAFs [117,118], while CAFs indirectly promote tumor growth and metastasis by recruiting immune cells, such as myeloid-derived suppressor cells (MDSCs) and regulatory T-cells (Tregs), through cytokines and chemokines, including IL-6, IL-8, and TGF-β1 [119]. Other TME components, including TAMs, MDSCs, and Tregs, further support tumor growth by suppressing anti-tumor CD8+ T-cells and NK cells [120].

CAFs also release stromal-derived factor 1 (SDF1), also known as C-X-C motif chemokine ligand 12 (CXCL12), which aids in recruiting endothelial progenitor cells (EPCs) to tumors. C-X-C chemokine receptor type 4 (CXCR4), expressed on cancer cells, binds to CXCL12 and promotes tumor growth [121]. Additionally, in several cancer types, CAFs express podoplanin, which correlates with the number of CD31+ blood vessels within tumors and VEGF-C expression in tumor cells. Interestingly, increased podoplanin expression in CAFs is associated with peri-tumoral microvessels and LYVE-1 positive lymphatic vessels, though it does not correlate with VEGF-A or VEGF-D expression in tumor cells [122].

CAFs significantly contribute to lymphangiogenesis and metastasis in the TME by activating Th2 T-cells, recruiting immunosuppressive cells, and releasing growth factors. However, the precise mechanisms remain incompletely understood. CAFs are instrumental in cancer development by reducing apoptosis and enhancing the proliferation, motility, and viability of cancer cells in close proximity to healthy cells [123]. They regulate cancer cell metabolism and growth by activating the autophagic pathway in response to oxidative stress. Moreover, CAFs can nourish cancer cells by producing cytokines and nutrients, such as ketones, which promote mitochondrial biogenesis and autophagy in nearby cancer cells [124]. CAF-derived cytokines and chemokines (e.g., CCL5, IL-6, and CXCL10) drive cancer cell metabolism by increasing the phosphorylation of phosphoglucomutase 1 and stimulating glycogen mobilization, NADPH synthesis, and the tricarboxylic acid (TCA) cycle, thereby supporting the growth and metastasis of ovarian cancer cells in vivo [125].

Recent advancements in cancer models, particularly 3D models, have provided insights into how tumor cells selectively control CAF functions. A study using organoid and mouse models of pancreatic ductal adenocarcinoma revealed that tumor-secreted ligands such as TGF-β and interleukin-1a (IL-1a) have opposing roles in producing two distinct CAF subtypes—myofibroblastic and inflammatory [126]. Inflammatory CAFs arise through IL-1a, leukemia inhibitory factor (LIF), Janus kinase (JAK), and signal transducers and activators of transcription (STAT) signaling, while TGF-β signaling blocks this process by enhancing myofibroblast differentiation and reducing IL-1 receptor type I (IL-1R1) expression [126]. 

The CAF classification system that emerged includes vascular CAFs (vCAFs), matrix CAFs (mCAFs), interferon-response CAFs (ifnCAFs), tumor-like CAFs (tCAFs), inflammatory CAFs (iCAFs), dividing CAFs (dCAFs), reticular-like CAFs (rCAFs), and antigen-presenting CAFs (apCAFs). This system was developed based on marker genes, biological functions, spatial distribution within the TME, and cellular interactions [127].

Innovative platforms, such as the tissue roll for the analysis of the cellular environment and response (TRACER), have enabled the study of tumor-stroma interactions in 3D systems. For example, co-culturing CAFs with FaDu cells (derived from hypopharyngeal squamous carcinoma) on the TRACER platform increased proliferation and invasive migration after 24 and 48 h of culture, although it did not affect radiation resistance [128]. Additionally, an in vitro organotypic microfluidic chip was used to investigate the interactions between tumor cells and CAFs by co-culturing breast cancer cells with patient-derived fibroblasts in 3D tumor and stroma zones. This 3D model revealed that CAFs accelerate breast cancer cell invasion and migration by inducing the expression of the glycoprotein nonmetastatic B (GPNMB) gene [129,130].

Pirfenidone (PFD) has shown anti-fibrotic and anti-inflammatory effects by downregulating TGF-β1, collagenase 1, IL-18, SDF1a, and bFGF. PFD can inhibit tumor cell growth mediated by CAFs, resulting in cell death in a 3D culture model of 4T1 tumor cells and CAFs. In vivo, PFD combined with doxorubicin can also inhibit lung metastasis and tumor growth [131]. Another approach to inhibiting CAFs involves using polyclonal rabbit anti-CAF antibodies obtained by immunizing rabbits with bFGF-activated fibroblasts. These polyclonal antibodies have been shown to slow tumor growth in murine models of CT26 colon cancer [132].

Inhibiting autophagy in CAFs presents another strategy for curbing cancer cell proliferation. Drugs such as metformin and gemcitabine have been reported to induce autophagy in CAFs. Chemotherapeutic agents, such as cyano-4-hydroxycinnamate (CHC) alone or in combination with metformin, have been shown to inhibit autophagic flux in CAFs and reduce tumor cell proliferation in both in vitro and syngeneic pancreatic cancer models, regardless of the chemotherapeutic agents used [133].

## 5. Interactions between Immune System, TME and Tumor Cells 

Clinical data from prognostic studies [134] demonstrate that lymphocytes can be highly effective in combating malignant cells. However, this effectiveness is not universal across all tumors. 

TAMs are known to produce proinflammatory molecules, including EGF, MMP9, MT1-MMP, MMP2, IL-1β, and TNFα [135]. Notably, in cancer patients, regulatory T-cells (Tregs) can suppress anti-tumor immune responses. Reducing Treg cells through antibodies or chemotherapy has been shown to enhance T-cell responses in patients undergoing immunotherapy [136]. Additionally, it has been observed that lactic acid in glycolytic tumors upregulates PD-1 on Treg cells, contributing to resistance to anti-PD-1 therapy in patients.

### 5.1. Dendritic Cells 

Dendritic cells (DCs) are the most potent antigen-presenting cells and are classified into several subsets [137]. They play a crucial role in tumor immunity by uptaking tumor antigens and presenting them to T-cells in draining lymph nodes, a critical step in initiating the anti-tumor immune response [137,138]. However, the significance of DCs in the tumor microenvironment (TME), their activation, and their role in tolerance induction is complex and sometimes contradictory. DCs are essential for T-cell-mediated cancer immunity as they regulate adaptive immune responses. Specifically, antitumor responses rely on a subset of conventional DCs that carry tumor antigens to the lymph nodes, activating cytotoxic T-cells. Although mature DCs provide costimulatory signals to T-cells, their ability to produce robust immunity is often compromised by inhibitory processes that affect DC maturation within tumors [139,140,141]. Overcoming these inhibitory pathways or directly activating DCs can enhance T-cell responses [142]. Despite limited clinical success, such as the FDA-approved DC vaccine for prostate cancer, therapeutic targeting of DCs holds promise in combination therapies [143].

DCs are naturally anti-tumorigenic, as they internalize and present antigens to T-cells in secondary lymphoid organs, bridging adaptive and innate immunity and initiating antigen-specific T-cell responses. However, the TME can alter DCs to support tumor progression. Tumor-associated DCs (TADCs) can release several growth factors and other molecules, including TGF-β, granulocyte-macrophage colony-stimulating factor (GM-CSF), CXCL12, and TNFα, which are all proangiogenic factors. Interestingly, VEGFs can also influence DCs by restricting their maturation through the suppression of NF-kB transcription. For example, an anti-VEGF-R3 antibody has been shown to prevent DC migration to draining lymph nodes in the eye [144]. Additionally, DCs can pick up antigens and migrate to draining lymph nodes via a CCL21 gradient, but certain tumor cells can exploit this mechanism by expressing the CCL21 receptor CCR7, enabling them to enter lymphatic capillaries [145].

Tumor cells also secrete TGF-β1, which induces the conversion of naïve T-cells into regulatory T-cells (Tregs). Tregs express CTLA-4, which interacts with CD80 and CD86 on antigen-presenting cells such as DCs, downregulating these co-stimulatory molecules and converting DCs into tolerogenic DCs [146]. These tolerogenic DCs within the TME then inhibit anti-tumor immunity by promoting the differentiation of Tregs, further suppressing the immune response against the tumor.

### 5.2. Macrophages 

Macrophages play a critical role in tumor hemangiogenesis and lymphangiogenesis [147]. TAMs respond to hypoxic conditions within tumor tissue by secreting various factors, including VEGFs, basic fibroblast growth factor (bFGF), thymidine phosphorylase [83], and multiple matrix metalloproteinases (MMPs), such as MMP-2, MMP-7, MMP-9, and MMP-12 [148]. TAMs can also transdifferentiate into lymphatic endothelial cells, expressing pro-lymphangiogenic factors such as VEGF-C, VEGF-D, and VEGF-R3 [149]. Additionally, TAMs have been reported to express the lymphatic vessel endothelial hyaluronan receptor 1 (LYVE-1) [150]. TAMs frequently exhibit an M2 phenotype, which is associated with immunosuppressive functions. For instance, in uveal melanoma, M2 macrophages, predominantly characterized by CD68+ CD163+ markers, are linked to poor prognosis [149]. Elevated levels of VEGF-C produced by TAMs are associated with increased peri-tumoral lymphatic vessel density in various cancers, including liver cancer and cutaneous squamous cell carcinoma [151]. Moreover, both TAMs and tumor cells secrete VEGF-A, VEGF-C, and MMP-9, which contribute to perineural lymphangiogenesis, further promoting tumor progression [152]. A study by Liu et al. [153] highlighted the potential for functional reprogramming of Kupffer cells (KCs), the largest population of hepatic macrophages, to treat liver cancer. The study utilized the CRISPR-Cas9 gene editing system to target specific genes in KCs, confirming their critical role in controlling tumor progression. The loss of KCs in metastatic tumors was found to correlate with a failure to control tumor growth, underscoring their importance in tumor immunity. TAMs also exert indirect proangiogenic effects by inhibiting the maturation of dendritic cells (DCs), which contributes to immunological tolerance and tumor progression [154]. An increased presence of immature DCs within tumor tissues is associated with enhanced tumor vascularization [155]. This inhibitory and immunosuppressive effect is primarily driven by interleukin 10 (IL-10), prostaglandin E2 (PGE2), and transforming growth factor-beta (TGF-β) secreted by TAMs [156]. 

### 5.3. T-Cells 

T lymphocytes are central to cellular immunity, acting as primary effector cells that release cytokines during immune responses to mediate inflammation and regulate other immune cells. Understanding cytokine regulation and T-cell function has paved the way for novel treatments across various human disorders, including cancer [157]. Recent advancements in research have enhanced our comprehension of adaptive immune cells’ roles within the tumor microenvironment (TME). Notably, the first therapeutic option aimed at stimulating T-cell functions, ipilimumab, was approved by the US FDA on 28 March 2011. However, tumor-specific CD8+ T-cells, when exposed to persistent antigenic stimulation, often enter a dysfunctional state known as “exhaustion”, leading to diminished effector function. Unfortunately, most patients exhibit limited efficacy to immune checkpoint inhibitors (ICIs) due to this exhaustion [158].

The TME imposes significant barriers on the metabolism and activity of tumor-infiltrating lymphocytes, with T-cells requiring substantial nutrient uptake to mount an effective immune response. Inadequate nutrient acquisition can severely impair effector T-cell differentiation and function [159]. Gamma delta (γδ) T-cells, known for their strong cytotoxic and pro-inflammatory activities, can kill a wide range of tumor cells, and the presence of tumor-infiltrating γδ T-cells is considered a positive prognostic marker in many studies [160]. Thus, they represent a promising target for cancer therapy. Conversely, regulatory T-cells (Tregs) are key immunosuppressive cells that promote tumor growth by inhibiting the effector immune response. Targeting Tregs, either alone or in combination with other immunotherapies, is being explored in clinical settings to enhance anti-tumor effects [161].

Tregs share signaling pathways with other T-cells and play a crucial role in maintaining immunological tolerance within the body. A significant challenge in cancer therapy is the difficulty in selectively blocking Tregs within the TME without disrupting self-tolerance [162]. Despite this, recent studies have focused on manipulating Treg cells as a therapeutic approach for liver cancer [163]. While immunotherapy has the potential to enhance anti-tumor immunity, its efficacy is often limited by the stromal cell barrier that restricts T-cell entry into the tumor [164,165]. Recent research by Mariathasan et al. [166] and Tauriello et al. [167] suggests that pre-existing immunity and tumor mutation burden are correlated with responses to anti-PD-L1 therapy. However, the presence of transforming growth factor-beta (TGF-β), particularly TGF-β1 secreted by tumor and stromal cells within the TME, is responsible for immune exclusion and resistance to anti-PD-L1 antibodies in metastatic cancers.

### 5.4. B-Cells 

Invading immune cells can significantly influence carcinogenesis by modulating cancer formation and anticancer responses. While many aspects of hepatocellular carcinoma (HCC)-related T lymphocytes have been extensively studied, the role of B lymphocytes remains less explored [168]. B cells are traditionally known for generating antibodies against target antigens. However, recent advances in B cell biology have revealed that B cells also secrete a wide range of cytokines and, like T helper cells, can be divided into subsets based on their cytokine profiles. One such subset, regulatory B cells (Bregs), has been discovered to play a critical role in maintaining the balance necessary for immune tolerance [169].

B lymphocytes are commonly found in draining lymph nodes, lymphoid structures surrounding the tumor microenvironment (TME), and at the invasive tumor border [170,171]. These cells play key roles in regulating tumor cell survival and proliferation and in the development of treatment resistance. Additionally, other studies have associated B cells with promoting immune escape mechanisms [172,173]. Due to the various underlying mechanisms, the precise role and impact of B cells in cancer development and tumor suppression remain critical areas of ongoing research [174,175,176].

Controlling B cells within the TME is essential for preventing cancer-induced immunosuppressive processes, such as the TGF-β-dependent conversion of FoxP3+ Treg cells, which support and promote metastasis [168,177,178]. In liver cancer patients, there is notably higher infiltration of TIM-1+ Breg cells in tumor tissue compared to peritumoral tissue. Tumor-derived exosomes have been shown to stimulate B cells, which then exert suppressive effects on CD8+ T-cells [158]. Moreover, B-cell infiltration in HCCs has been associated with longer patient survival and a unique immunoglobulin profile, which correlates with improved patient outcomes. B lymphocytes contribute to local tumor control by secreting higher levels of anticancer immunoglobulins [179].

### 5.5. Neutrophils 

Neutrophils play a crucial role in the early immune response to infections through mechanisms such as phagocytosis and the formation of extracellular traps (NETs). These cells enhance the inflammatory response by producing cytokines and help resolve inflammation by phagocytizing dead cells, presenting antigens, and thus contributing to the termination of the inflammatory process [180]. However, in the context of cancer, neutrophils can adopt pro-tumoral roles by secreting factors such as MMP9, HGF, and VEGF, which promote angiogenesis. In fact, reducing neutrophil numbers has been shown to halt the angiogenic switch, a critical step in tumor progression, which is why inhibiting IL-8 can slow tumor growth [181]. 

In solid tumors such as liver cancer, neutrophil infiltration is typically linked to poor prognosis. A higher neutrophil-to-lymphocyte ratio in the bloodstream is often indicative of worse outcomes in various cancers [182,183]. The dominance of neutrophils over macrophages in tumors is largely driven by chemokines such as IL-8, produced by tumor cells, inflammation, and necrosis. Despite their transient nature, neutrophils can impact tumor progression in two distinct ways. They can function as anti-tumor neutrophils (N1 neutrophils), particularly in the presence of IL-12 and TNF-α, with CD8+ T-cells playing a crucial role in this activity. The dynamic behavior of neutrophils within the tumor microenvironment highlights their complex and multifaceted roles in cancer progression and immune responses [181,184].

### 5.6. Eosinophils 

Eosinophils, a subset of white blood cells, play a crucial role in the immune system by defending vertebrates against multicellular parasites and certain infections [185]. Within the tumor microenvironment (TME), eosinophils secrete a range of soluble mediators and effector molecules that can have significant immunoregulatory effects. As such, eosinophils are considered potent immune effectors and modulators within the TME [186]. Although eosinophil infiltration in tumors has been recognized for some time, their role in the TME has only recently gained significant attention [187]. Upon infiltrating tumors, eosinophils exert pleiotropic effects through at least two non-exclusive mechanisms: complex crosstalk with lymphocytes and direct interactions with tumor cells [188]. Eosinophils are highly responsive to various stimuli and can rapidly release soluble mediators that not only accelerate tumor growth but also promote angiogenesis and matrix remodeling, further influencing tumor progression [189].

### 5.7. Mast Cells 

Mast cells (MCs) play a pivotal role in the interaction between inflammatory and tumor cells by producing both conventional and unconventional proangiogenic mediators, which are crucial in regulating tumor angiogenesis. The density of MCs in human malignancies is closely correlated with tumor angiogenesis [190]. One of the most potent angiogenic mediators released by MCs is tryptase, a protease that stimulates human vascular endothelium and promotes tumor cell proliferation, invasion, and metastasis in a paracrine manner. Given its significant proangiogenic activity, tryptase has been proposed as a promising therapeutic target in cancer treatment [191]. Beyond their role in angiogenesis, MCs significantly influence both innate and adaptive immune responses. They express Toll-like receptors (TLRs) 1 through 7, 9, and Fc receptors, which are involved in mucosal barrier immune defense. Additionally, MCs attract various immune cells, including neutrophils, eosinophils, CD8+ T-cells, and natural killer lymphocytes (NK LTs), by releasing inflammatory mediators or cytokines from their granules. MCs also play a role in stimulating dendritic cells (DCs), presenting antigens via MHC class I or II molecules, and supporting angiogenesis [192].

Typically, MCs are tissue-resident innate immune cells that regulate inflammation and homeostasis. However, they proliferate within the tumor stroma of various human cancers. The impact of MC density on prognosis varies depending on the tumor type and stage; higher MC density can be associated with either favorable or poor outcomes, as MCs modulate cell proliferation, survival, angiogenesis, invasiveness, and metastasis within the tumor microenvironment (TME) [193,194]. Tumor-associated mast cells influence the TME through interactions with invading cells [195]. Although the significance of MCs in the TME is increasingly recognized, their exact role as modulators within the TME remains unclear and is a subject of ongoing research [196].

### 5.8. Natural Killer Cells

Natural killer (NK) cells are cytotoxic lymphocytes with the innate ability to recognize and eliminate tumor cells, making them a promising candidate for cancer therapy. The adoptive transfer of autologous or allogeneic NK cells has emerged as a potential therapeutic approach. However, the tumor microenvironment (TME) can impair NK cell function, phenotype, activation, and persistence, leading to their exhaustion [197,198]. To counteract these challenges, activation strategies using cytokines or their analogs have been tested to enhance NK cell persistence, activation, and cytolytic activity [199]. Additionally, the incorporation of chimeric antigen receptors (CARs) has improved the targeting selectivity of NK cells, while checkpoint blockade has shown promise in rejuvenating NK cell function [199]. 

Significant efforts are being made to fully exploit the anti-tumor potential of NK cells in clinical settings, given their role as potential effector cells in cell-based cancer immunotherapy [200]. Various approaches include large-scale NK cell expansion for adoptive transfer, creating a supportive environment for NK cell activity, redirecting NK cells against tumor cells, and overcoming inhibitory signals that limit NK cell effectiveness [201]. 

Immune escape is a hallmark of cancer, contributing to tumor progression and metastasis [202]. NK cells, as key effector cells of innate immunity, are highly heterogeneous within their microenvironment, making them crucial targets for therapeutic intervention [203]. Most current immunotherapies focus on enhancing T-cell immunity by modulating inhibitory signals; however, the limited success of T-cell-based therapies underscores the need for alternative approaches, such as utilizing NK cells. Tumors can adapt to resist NK cell-induced cytotoxicity, but cytokine supplementation, blockade of suppressive molecules, and genetic engineering of NK cells are showing promise in both solid and hematological malignancies [204]. 

Furthermore, the development of CAR NK cells, which redirect NK cells to target tumor cells expressing specific antigens, has significantly advanced cancer treatment options [205]. CAR NK cells hold the potential to be used as universal CAR cells without the need for matching or prior exposure to tumor-associated antigens. Recent clinical trials have demonstrated the feasibility of CAR NK cells as “off-the-shelf” anti-cancer immunotherapies, offering new avenues for treatment [205].

## 6. Conclusions

Future perspectives on understanding and targeting the TME in liver cancer could revolutionize cancer therapy. As our knowledge deepens, it becomes clear that the TME is not just a passive bystander but an active participant in cancer progression, influencing tumor growth, immune evasion, and metastasis. Future research should focus on unraveling the intricate interactions between tumor cells and their microenvironment, particularly in the context of hypoxia, immune suppression, and metabolic reprogramming. Innovative therapeutic strategies that target both tumor cells and the TME, such as modulating immune responses or disrupting metabolic symbiosis, hold great promise. Additionally, developing precise imaging techniques to monitor TME dynamics and integrating these with personalized treatment approaches could lead to more effective, tailored therapies. By targeting the TME alongside tumor cells, we may significantly improve treatment outcomes and offer new hope for patients with liver cancer.

## Data Availability

All data generated or analyzed during this study are included in this article and will be available upon reasonable request.

## Abbreviations

α-SMA: Alpha-smooth muscle actin; Ang2: Angiopoietin-2; ATG5: Autophagy-related gene 5; bFGF: Basic fibroblast growth factor; BMP1: Bone morphogenic protein 1; CAF: Cancer-associated fibroblasts; CARs: Chimeric antigen receptors; CCL5: Chemokine ligand 5; CHC: Cyano-4-hydroxycinnamate; CSC: Cancer stem cell; CXCL: Chemokines; CXCL10: C-X-C motif chemokine 10; CXCL12: C-X-C motif chemokine ligand 12; CXCR4: CXC-chemokine receptor 4; DCs: Dendritic cells; ECM: Extracellular matrix; EMT: Epithelial-to-mesenchymal transition; FAP: Fibroblast-activating protein; FASL: Fas ligand; FGF: Fibroblast growth factor; GPNMB: Glycoprotein nonmetastatic B; GF: Growth factor; GM-CSF: Granulocyte-macrophage colony-stimulating factor; GPC-3: Glypican-3; HCC: Hepatocellular carcinoma; HGF: Hepatocyte growth factor; HIFs: Hypoxia-inducible factor; HNSCC: Head and neck squamous cell carcinoma; ICC: Intrahepatic cholangiocarcinoma; ICIs: Immune checkpoint inhibitors; IDO: Indolamine 2,3-dioxygenase; KCs: Kupffer cells; LAMP2: Lysosome-associated membrane protein 2; MCs: Mast cells; M2: M2 macrophage; MDSC: Myeloid-derived suppressor cell; MMP: Matrix metalloproteinase; NADPH: Nicotinamide adenine dinucleotide phosphate; N2: Neutrophil; NETs: Extracellular trapping; NK: Natural killer; NKT: Natural killer T-cells; PD-1: Programmed cell death protein 1; PDGF: Platelet-derived growth factor; PD-L1: Programmed cell death ligand-1; PFD: Pirfenidone; PGE2: Prostaglandin E2; PLC: Primary liver cancer; ROS: Reactive oxygen species; TAM: Tumor-associated macrophages; TGF-β: Transforming growth factor-beta; TIM3: T-cell immunoglobulin domain and mucin domain 3; TME: Tumor microenvironment; TNC: Tenascin-C; TNBC: Triple negative breast cancer; Treg: T-regulatory lymphocyte; VEGF: Vascular endothelial growth factor.
